# Bilateral Collicular Interaction: Modulation of Auditory Signal Processing in Amplitude Domain

**DOI:** 10.1371/journal.pone.0041311

**Published:** 2012-07-24

**Authors:** Hui-Xian Mei, Liang Cheng, Jia Tang, Zi-Ying Fu, Xin Wang, Philip H.-S. Jen, Qi-Cai Chen

**Affiliations:** 1 College of Life sciences and Hubei Key Lab of Genetic Regulation and Integrative Biology, Central China Normal University, Wuhan, Hubei, China; 2 Division of Biological Sciences, University of Missouri-Columbia, Columbia, Missouri, United States of America; University of Salamanca- Institute for Neuroscience of Castille and Leon and Medical School, Spain

## Abstract

In the ascending auditory pathway, the inferior colliculus (IC) receives and integrates excitatory and inhibitory inputs from many lower auditory nuclei, intrinsic projections within the IC, contralateral IC through the commissure of the IC and from the auditory cortex. All these connections make the IC a major center for subcortical temporal and spectral integration of auditory information. In this study, we examine bilateral collicular interaction in modulating amplitude-domain signal processing using electrophysiological recording, acoustic and focal electrical stimulation. Focal electrical stimulation of one (ipsilateral) IC produces widespread inhibition (61.6%) and focused facilitation (9.1%) of responses of neurons in the other (contralateral) IC, while 29.3% of the neurons were not affected. Bilateral collicular interaction produces a decrease in the response magnitude and an increase in the response latency of inhibited IC neurons but produces opposite effects on the response of facilitated IC neurons. These two groups of neurons are not separately located and are tonotopically organized within the IC. The modulation effect is most effective at low sound level and is dependent upon the interval between the acoustic and electric stimuli. The focal electrical stimulation of the ipsilateral IC compresses or expands the rate-level functions of contralateral IC neurons. The focal electrical stimulation also produces a shift in the minimum threshold and dynamic range of contralateral IC neurons for as long as 150 minutes. The degree of bilateral collicular interaction is dependent upon the difference in the best frequency between the electrically stimulated IC neurons and modulated IC neurons. These data suggest that bilateral collicular interaction mainly changes the ratio between excitation and inhibition during signal processing so as to sharpen the amplitude sensitivity of IC neurons. Bilateral interaction may be also involved in acoustic-experience-dependent plasticity in the IC. Three possible neural pathways underlying the bilateral collicular interaction are discussed.

## Introduction

In sound reception, signal processing in higher centers of the auditory pathway is based on neural interactions from divergent and convergent projections through the interplay of excitation and inhibition [1]. For example, the central nucleus of the inferior colliculus (IC) receives and integrates excitatory and inhibitory inputs from many bilateral lower auditory nuclei as well as from the auditory cortex [2]–[11]. Neurons in one IC also receive projections within the IC and from the contralateral IC through the commissure of the IC [12]–[17]. For this reason, many studies have examined the interplay of excitation and inhibition in shaping the temporal processing and multiple-parametric selectivity in the IC [18]–[22]. Other studies have shown that the massive descending corticofugal system not only adjusts and improves ongoing collicular auditory signal processing in multiple-parametric domains but also reorganizes collicular auditory maps according to the acoustic experience [23]–[38].

Besides these numerous studies of the interplay of excitation and inhibition in afferent and efferent inputs to the IC, others have been devoted to examining the interaction between collicular neurons within the same IC and between the two ICs regarding auditory signal processing [15], [39]–[42]. For example, when two neurons at different depths within the same IC are recorded under two-tone stimulation conditions, interaction between the two IC neurons produces inhibition (82%) and facilitation (18%) of the response of affected IC neurons. This colliculo-collicular interaction also sharpens the excitatory frequency tuning curves and decreases the rate-level function (RLF) of inhibited IC neurons through GABAergic inhibition [39], [41], [42]. Another study shows that focal electrical stimulation of collicular neurons evokes BF shifts of collicular neurons located near the stimulated ones and the collicular BF shifts depend on corticofugal feedback [37]. The collicular BF shift also depends on acetylcholine because it has been demonstrated that atropine (an antagonist of muscarinic acetylcholinergic receptors) applied to the IC blocks the development of collicular BF shifts [43].

**Figure 1:**
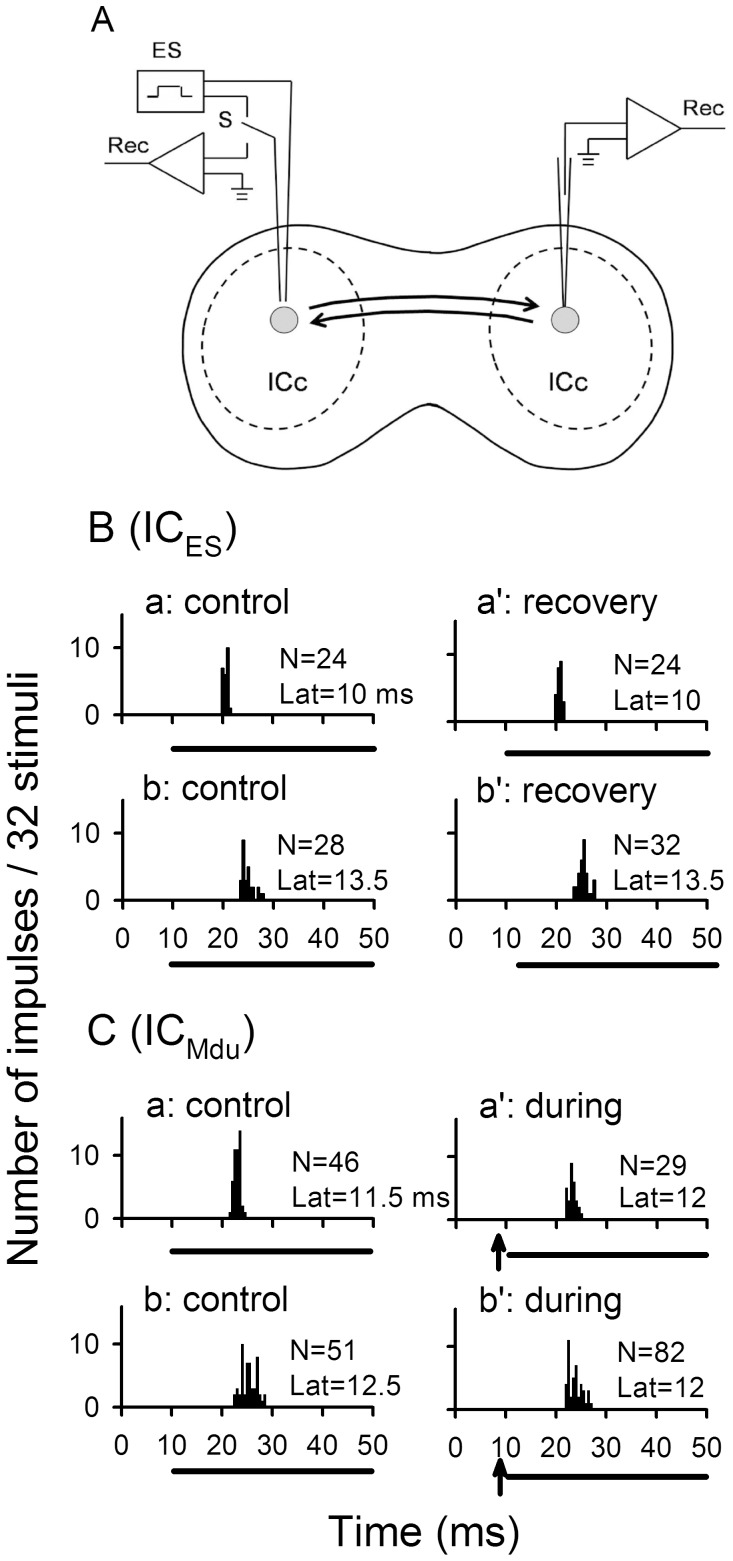
Experimental arrangement and responses of IC_ES_ and IC_Mdu_ neurons under different stimulation conditions. A: A schematic drawing of a coronary section through the inferior colliculi (ICs) of mice (*Mus musculus,* Km).The dashed lines delimit the central nuclei of the IC and its surrounding cortices. Filled grey circles indicate IC neurons which are bilaterally interconnected by the fibre projection (solid line) of the commissure of the IC. The drawing also shows the experimental arrangement for focal electrical stimulation and recording of the response of a neuron in one IC with a pair of custom-made tungsten electrodes (left) and recording of the response of a neuron in the other IC with a 2 M NaCl glass electrode (right). B: Peri-stimulus-time (PST) histograms showing the responses of two representative IC_ES_ neurons obtained before and after recovery from self focal electrical stimulation (a vs a’, b vs b’). C: PST histograms of inhibited (a vs a’) and facilitated (b vs b’) IC_Mdu_ neurons obtained before and during focal electrical stimulation of IC_ES_ neurons (abbreviated as IC_ES_ focal electrical stimulation). All PST histograms were obtained with a best frequency (BF) sounds delivered at 10 dB above the minimum threshold (MT). N: number of impulses in each PST histogram. Lat: response latency. Horizontal bar: acoustic stimulus. Arrows: focal electrical stimulus. The BF (kHz), MT (dB SPL) and recording depth (µm) of these four IC neurons were 11.3, 68, 740 (Ba); 14.1, 58, 859 (Bb); 15, 59, 1114 (Ca); 9.8, 71, 1378 (Cb).

Other studies examined the bilateral collicular interaction in signal processing by comparing the sound-evoked responses of one IC neuron before and after hydraulic injection of kynurenic acid (antagonist of glutamatic acid) into the corresponding region of the other IC [15], [40]. They indicate that the bilateral collicular interaction is mediated through the commissure of the IC to modulate the shape of the frequency response area, number of impulses and the shape of the RLFs of IC neurons. However, these studies did not determine if the degree of bilateral collicular interaction was related to the response parameters of neurons such as the best frequency, minimum threshold and latency of the neurons in the two ICs.

The main objective of this study is to examine the interaction of collicular neurons between the two ICs in amplitude-domain signal processing using electrophysiological recording, acoustic and focal electrical stimulation. Specifically, we study the effect of bilateral collicular interaction on amplitude sensitivity in relation to the tonotopy and plasticity in one IC during and after focal electrical stimulation of the other IC.

**Table 1:** The recording depth, BF, MT and latency of IC_Mdu_ neurons whose responses were inhibited or facilitated during IC_ES_ electrical stimulation.

		Depth(µm)	BF(kHz)	MT(dB SPL)	Latency (ms)
Inhibition	Range	227∼2003	5.5∼27.6	15∼87	8.5∼23.5
n = 61	mean±S.D.	1083.2±401.1	14.2±4.8	54±17.6	14.9±4.0
Facilitation	Range	390∼1378	8.5∼19.6	56∼75	10.0∼18.0
n = 9	mean±S.D.	1046.1±304.6	11.6±3.6	65±6.1	13.3±2.5
*t* test, *p*		>0.05	>0.05	>0.05	>0.05

**Figure 2:**
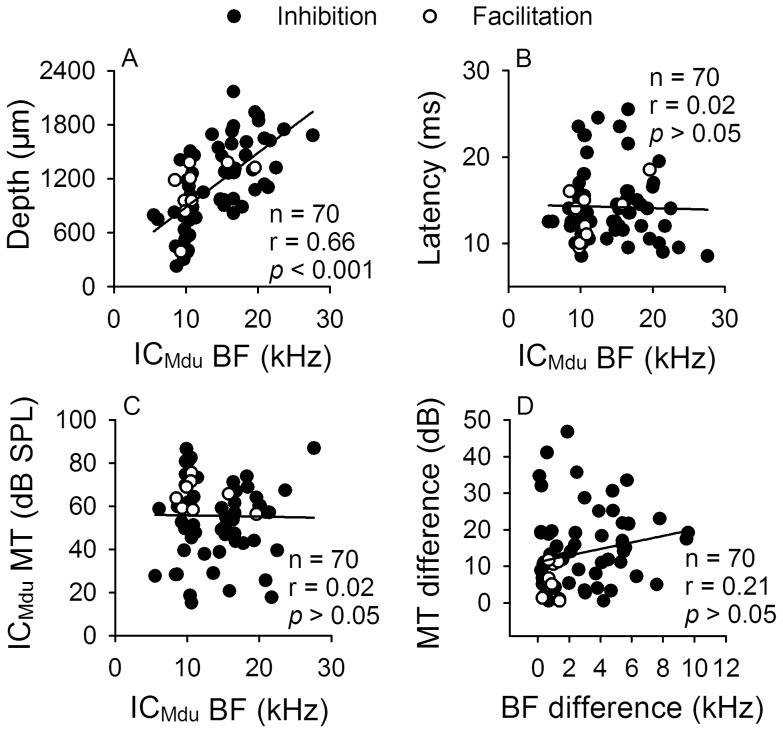
Correlation among different parameters of IC_Mdu_ neurons. Scatter plots showing the distribution of the BFs of inhibited and facilitated IC_Mdu_ neurons against recording depth (A), latency (B) and MT (C) as well as the BF difference against MT difference (D). Within each plot, the linear regression line and correlation coefficient are shown with a solid line and r. *p*: significance level.

## Methods

### Animal Preparation and Surgery

A total of 21 (8 females, 13 males; body weight, b.w. 20–25 g) adult mice (*Mus musculus,* Km) (2–3 months, supplied by the Center for Disease Control and Prevention of Hubei Province in China) was used for this study. All experiments were conducted with the approval of the Institutional Animal Care and Use Committee of Central China Normal University, Wuhan, Hubei, China. The surgical procedures for recording of sound-activated responses were basically the same as described in previous studies [44], [45]. Briefly, the flat head of a 2.0-cm nail was glued onto the exposed skull of each Nembutal anesthetized mouse (60–90 mg/kg b.w.) with acrylic glue and dental cement. Exposed tissue was treated with an antibiotic (Neosporin) to prevent inflammation. After 1–2 hours of post-surgery, the anesthetized animal was tied to a metal plate inside a custom-made, double-wall, sound-proof room (temperature 28°–30°C). The ceiling and inside walls of the room were covered with 2-cm polyurethane foam to reduce echoes.

**Figure 3:**
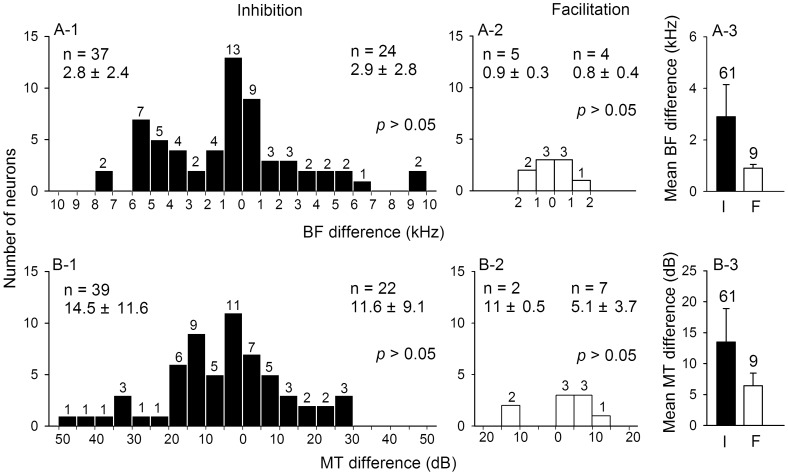
BF and MT differences of inhibited and facilitated IC_Mdu_ neurons. Distribution histograms showing the BF (kHz)(A) and MT (dB)(B) differences of inhibited (A-1,B-1) and facilitated (A-2,B-2) IC_Mdu_ neurons. Numbers in the right abscissa indicate that IC_Mdu_ neurons had larger BF and MT than IC_ES_ neurons. The opposite is shown in the left abscissa. The mean and standard deviation of each group of neurons (n) are shown. *p*: significance level of *t* test. A-3,B-3: the average BF and MT differences of inhibited and facilitated IC_Mdu_ neurons. The number of neurons and half a standard deviation are shown atop of each bar.

After fixing the head with a set screw and orienting the eye-nostril line to 0° in azimuth and 0° in elevation of the frontal auditory space, small holes (diameter: 200–500 µm) were bored in the skull above each IC for orthogonal insertion of custom-made tungsten electrodes (see below) and 2 M NaCl glass pipette electrode (tip diameter: <1 µm, impedance: 5–10 MΩ) for focal electrical stimulation and for recording sound-activated responses in the central nucleus of the IC. The depths of recorded IC neurons were read from the scale of two microdrives (David-Kopf, model 640, USA). A common indifferent electrode (silver wire) was placed at the nearby temporal muscles. Additional doses of anesthetics (one fourth of original) were administered during later phases of recording when the animal showed signs of discomfort as judged by increasing respiration and minor movement of limbs. In addition, a local anesthetic (Lidocaine) was applied to the open wound area to reduce any possible pain. When the animal was in good physiological condition, it was used up to 3 recording sessions on separate days, and each recording session typically lasted 2–6 hours to minimize the number of animals used for this study. Between recording sessions, the scalp was treated with antibiotic cream (Neosporin) to prevent inflammation and the skin was stitched back to the normal position before being put into the cage of animal room. The animal was then fed with food and water ad libitum until the next experimental session.

**Figure 4:**
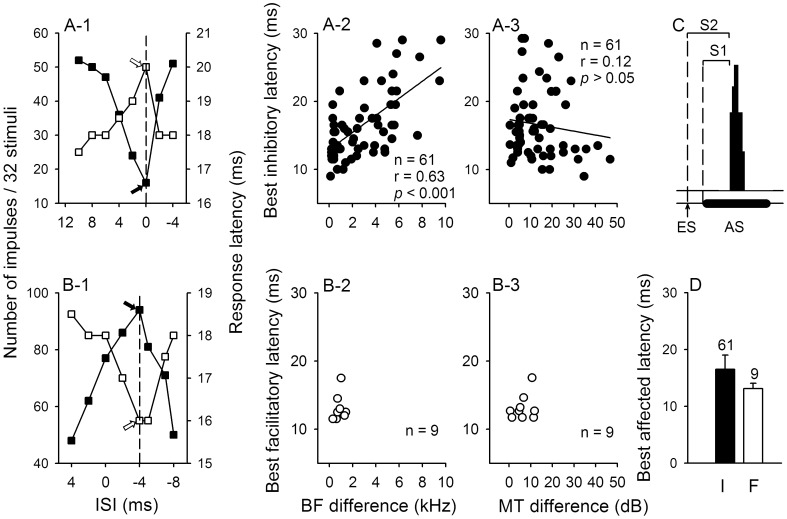
Variation of modulation of IC_Mdu_ neurons with inter-stimulus interval and BF and MT differences. Variation in the number of impulses (solid squares refer to left ordinates) and the response latency (unfilled square refer to right ordinates, S1 in C) of two IC_Mdu_ neurons during IC_ES_ focal electrical stimulation at each inter-stimulus interval (ISI in ms). At each ISI, there was an inhibitory (A-1) or facilitatory (B-1) latency (S2 in C). The smallest number of impulses (solid arrow) and the longest acoustic response latency (unfilled arrow) were always obtained at the best inhibitory latency for the inhibited IC_Mdu_ neuron but the opposite were obtained at the best facilitatory latency for the facilitated IC_Mdu_ neuron. The BF (kHz), MT (dB SPL) and recording depth (µm) of these two IC_Mdu_ neurons were 9.8, 64, 634 (Aa); 9.3, 59, 390 (Ab). A-2,A-3,B-2,B-3: The scatter plots showing the best inhibitory latency (solid circles, n = 61) and the best facilitatory latency (unfilled circles, n = 9) of IC_Mdu_ neurons in relation to BF and MT differences between IC_ES_ and IC_Mdu_ neurons. A linear regression line and correlation coefficient are shown with a solid line and r. *p*: significance level. C: A sketch showing the PST histogram of a hypothetical IC_Mdu_ neuron in response to acoustic stimulus (AS) combined with IC_ES_ electrical stimulation (ES). S1: the acoustically activated response latency; S2: the inhibitory or facilitatory latency expressed as the time interval between the onset of ES and auditory response. D: the mean best affected latency of 61 inhibited (I) and 9 facilitated (F) IC_Mdu_ neurons. The number of neurons and half of a standard deviation are shown atop of each bar.

### Stimulation and Isolation of Acoustically Activated Collicular (IC) Neurons

For acoustic stimulation, continuous sine waves from a function generator (GFG-8016G, Good Will Inst Co., Ltd, Bayan Lepas, Penang, Malaysia) were formed into 40 ms pure tones (5 ms rise-decay times) with a custom-made tone burst generator (electronic switch) driven by a stimulator (Model SEN-7203, Nihon Kohden Co, Shinjuku, Tokyo, Japan) and delivered at 2 pulses per second. The tone pulses were then amplified (custom-made amplifier) after passing a decade attenuator (LAT-45, Leader, Kohokuku, Yokohama, Japan) before they were fed into a small loudspeaker (AKG model CK 50, 1.5 cm in diameter, 1.2 g, frequency response 1–100 kHz). The loudspeaker was calibrated with a 1/4-inch microphone (4939, B&K, Denmark) placed at the mouse’s ear using a measuring amplifier (2610, B&K, Denmark). The output of the loudspeaker was expressed in decibel sound pressure level (dB SPL) in reference to 20 µPa root mean square. A frequency response curve of the loudspeaker was plotted to determine the maximal available sound amplitude at each frequency. The maximal stimulus level ranged from 95 to 120 dB SPL between 10 and 80 kHz but dropped off sharply to 80 dB SPL at 100 kHz thereafter.

**Figure 5:**
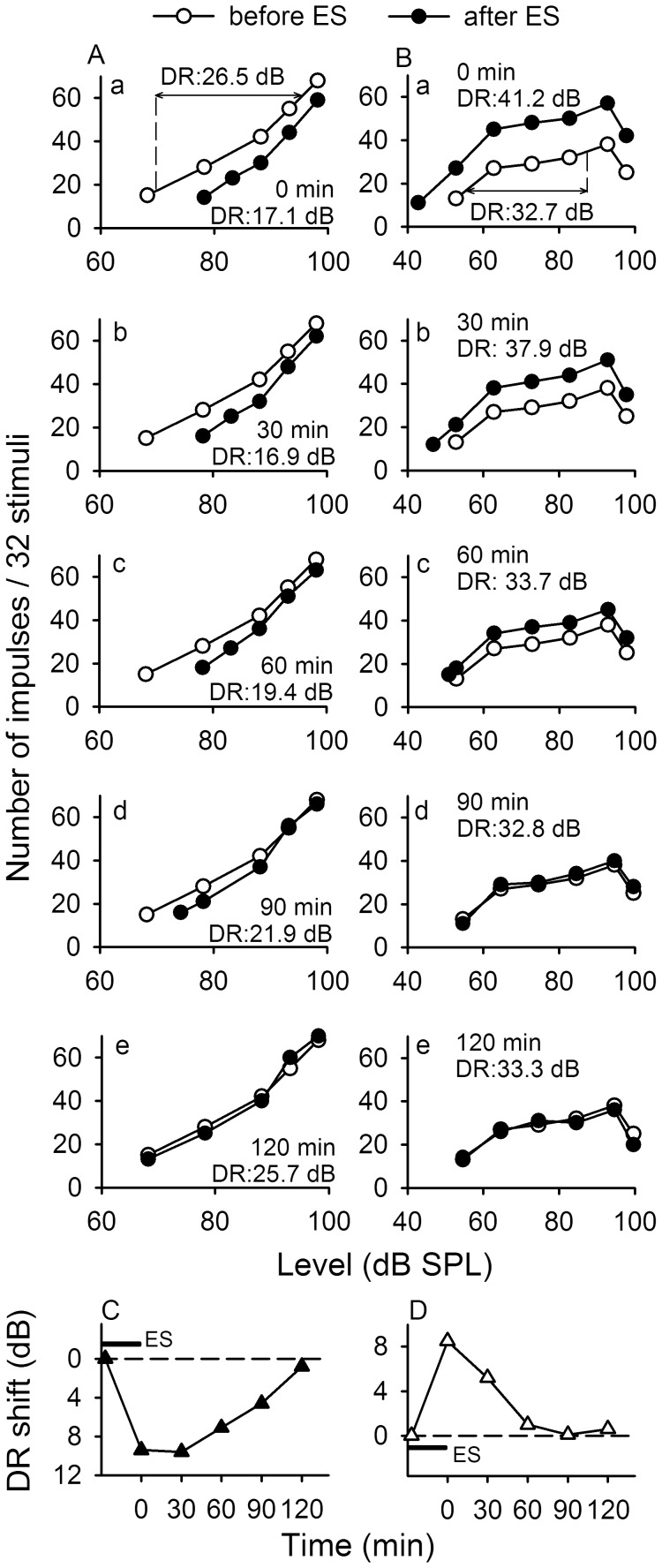
Modulation of the rate-level function of IC_Mdu_ neurons after 30 minute IC_ES_ focal electrical stimulation. The rate-level function (RLF) of an inhibited (A) and a facilitated (B) IC_Mdu_ neuron measured before (unfilled circles) and at different times (at 0 min, a; 30 min, b; 60 min, c; 90 min, d; 120 min, e, filled circles) after 30 minute IC_ES_ focal electrical stimulation. The dynamic range (DR) of the control RLF (unfilled circles) and modulated RLF (filled circles) are shown. C,D: The time course of DR shift of these two IC_Mdu_ neurons after 30 minute IC_ES_ focal electrical stimulation (indicated with short horizontal bar). Downward curve(C) indicates DR is decreased, while upward curve (D) indicates the opposite. The BF (kHz), MT (dB SPL) and recording depth (µm) of these two IC_Mdu_ neurons were 16.9, 68, 1205 for A and 12.2, 53, 954 for B.

Two insulated tungsten electrodes (FHC Inc, Bodowin, ME, USA) were glued together (tip: <10 µm, inter-tip distance: ≤100 µm) to form a pair of tungsten electrodes. These electrodes were used for recording sound-activated responses of IC neurons and for focal electrical stimulation in the IC recording site (4 ms train of four monophasic pulses of 0.1 ms with 0.9 pulse-gap at 2 train/s, 5–50 µA) using stimulator (Model SEN-7203, Nihon Kohden CO, Tokyo, Japan) and stimulus isolation unit (Model Nihon Kohden CO, Tokyo, Japan)(Fig. 1A, left).

**Figure 6:**
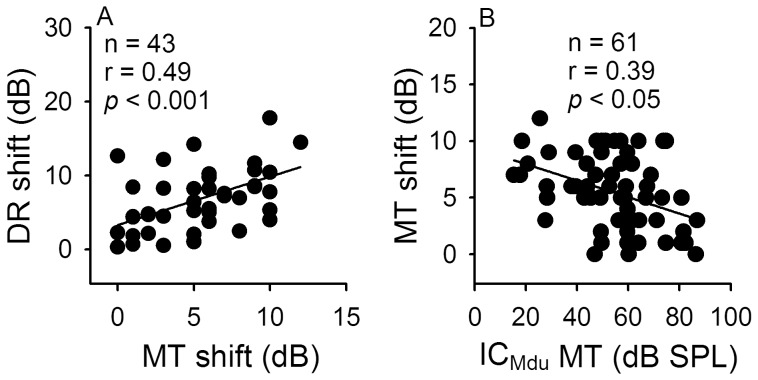
Correlation among DR and MT shifts and the MT of IC_Mdu_ neurons. Scatter plots of the MT shift against DR shift and MT of IC_Mdu_ neurons. N: number of IC_Mdu_ neurons. The linear regression line and correlation coefficient are shown with a solid line and r. *p*: significance level.

During experiment, a 40 ms sound was delivered (at 2 pulses/s) from the loudspeaker placed 30 cm away from the animal and 60° contralateral to the recording site in order to maximally excite the recorded IC neuron [44], [45]. When an IC neuron was isolated (the first IC neuron, abbreviated as the IC_ES_ neuron) with a pair of custom-made tungsten electrodes, its best frequency (BF) and minimum threshold (MT) were audio-visually measured by systematically changing the frequency and level of sound pulses. The sound frequency that elicited the neurons’ response at the lowest amplitude was defined as the BF. The threshold at the BF was defined as the MT. At the MT, the neuron, on average, responded with 50% probability to BF pulses.

**Figure 7:**
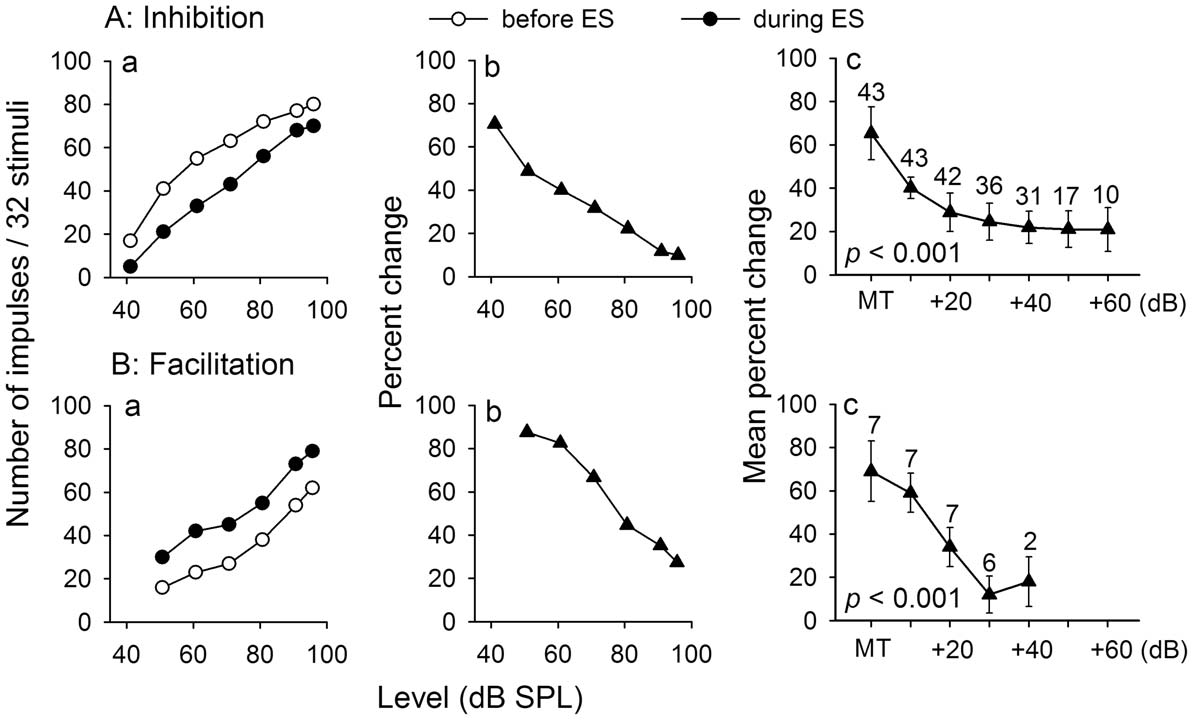
Level-dependent modulation of IC_Mdu_ neurons during IC_ES_ focal electrical stimulation. The RLF of inhibited (Aa) and facilitated (Ba) IC_Mdu_ neurons obtained before (unfilled circles) and during (filled circles) IC_ES_ focal electrical stimulation. Ab,Bb: The percent change in the number of impulses of these two IC_Mdu_ neurons with sound level during IC_ES_ focal electrical stimulation. The BF (kHz), MT (dB SPL) and recording depth (µm) of these two IC_Mdu_ neurons were 16.6, 41, 1207 (A); 15.7, 51, 1051 (B). Ac, Bc: Mean percent change in the number of impulses of IC_Mdu_ neurons measured at MT and at 10 dB increments above MT of each neuron. The number of IC_Mdu_ neurons measured at each point is shown. *p*: significance level. The vertical bar: half of a standard deviation. Note that percent change significantly decreased with sound level (one-way ANOVA, *p*<0.001).

Acoustically activated responses of an IC neuron in the other IC (the second IC neuron, abbreviated as the IC_Mdu_ neuron) was then isolated with a 2 M NaCl glass electrode after moving the loudspeaker 60° contralateral to the isolated IC_Mdu_ neuron (Fig. 1A, right). After determining its BF and MT, its response to BF sound pulses delivered at 10 dB above the MT was recorded as the control response. The neuron’s response was then monitored again during focal electrical stimulation in the IC_ES_ neuron (hereafter abbreviated as IC_ES_ focal electrical stimulation) through the custom-made tungsten electrodes. Electrical stimulus was synchronized with the acoustic stimulus by a synchrony trigger signal (2 pulses/s) from the stimulator (Model SEN-7203, Nihon Kohden Co, Shinjuku, Tokyo, Japan) which triggered the custom-made tone burst generator and an electric stimulator such that the interval between the two stimuli could be adjusted at random. At first, the electrical stimulation was delivered at 2 trains/s between 5 and 50 µA and at a randomly chosen inter-stimulus interval (ISI). The current level was gradually increased in order to find an IC_Mdu_ neuron affected by the IC_ES_ electrical stimulation and to observe the effect on response of the IC_Mdu_ neuron under different current level. Then, the electrical stimulation current was fixed at moderate level (25 µA) and the ISI was adjusted systematically to determine the optimal ISI that produced maximal effect. If the percent change in number of impulses of the IC_Mdu_ neuron induced by the focal electrical stimulation didn’t reach 30%, the IC_Mdu_ neuron was abandoned. Otherwise it was regarded as a modulated IC_Mdu_ neuron. At the optimal ISI, the response latency and RLF of the modulated IC_Mdu_ neuron were then measured before and during IC_ES_ focal electrical stimulation. The response latency was defined as the interval between the onset of the acoustic stimulus and the neuronal response. A RLF was measured with a neuron’s number of impulses obtained with a BF sound delivered at MT and at 10 dB increments above the MT.

**Figure 8:**
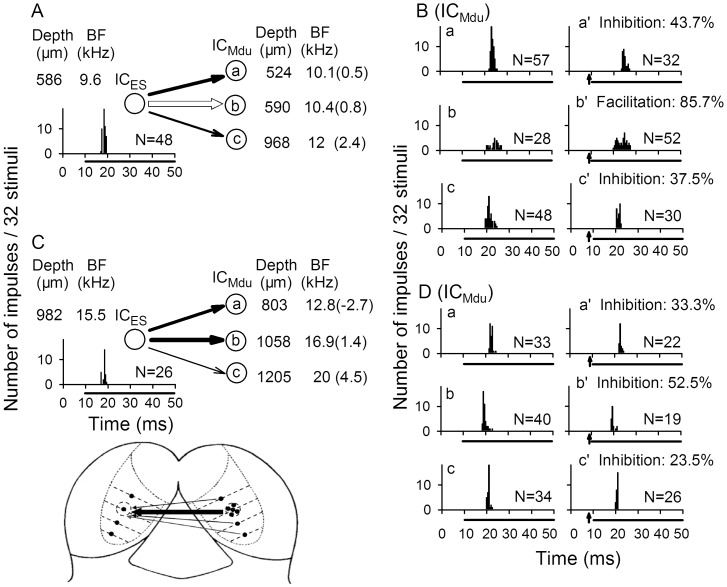
Modulation of IC_Mdu_ neurons during IC_ES_ focal electrical stimulation. Focal electrical stimulation of one IC_ES_ neuron produced inhibition of two IC_Mdu_ neurons and facilitation of one IC_Mdu_ neuron (A,B). Focal electrical stimulation of another IC_ES_ neuron produced inhibition of all three IC_Mdu_ neurons (C,D). The PST histogram, number of impulses (N), recording depth and BF of all IC_ES_ and IC_Mdu_ neurons are shown. %: percent inhibition or facilitation of IC_Mdu_ neurons. Arrow: IC_ES_ focal electrical stimulation. Bottom: a carton showing the divergent pattern of connections from an injection site of one IC through the commissure of the IC to different frequency laminae of the other IC (adapted from [51]).

As in previous studies [23]–[25], [31], [38], the modulation of the response of IC_Mdu_ neurons disappeared upon the cessation of IC_ES_ focal electrical stimulation when delivered at 2 trains/s at 25 µA. Therefore, to study the plasticity of the responses of IC_Mdu_ neurons, IC_ES_ focal electrical stimulation was delivered at the optimal ISI and 10 trains/s for 30 minutes synchronized with the onset of acoustic stimulus (the BF of IC_ES_ neuron delivered at 10 dB above its MT). The discharge pattern and the RLF of the IC_Mdu_ neuron were then progressively monitored at 0, 30, 60, 90, 120, 150 minutes after 30 minute IC_ES_ focal electrical stimulation.

**Figure 9:**
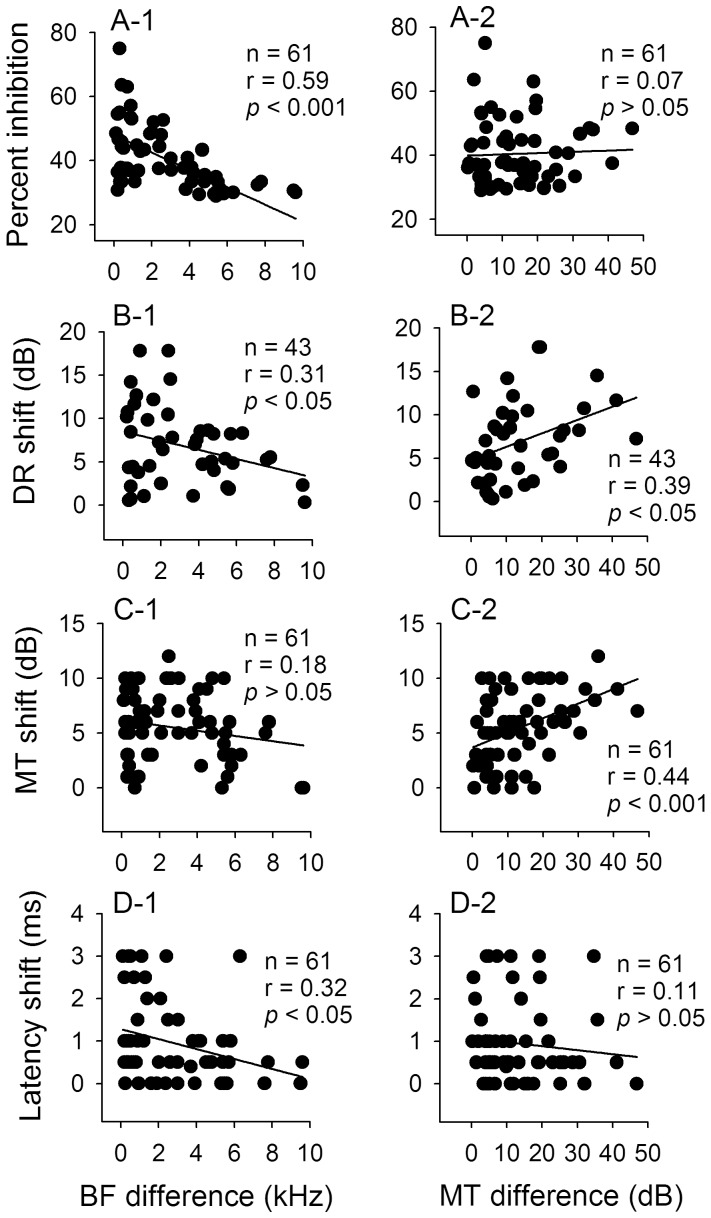
Correlation of different modulated parameters of IC_Mdu_ neurons in relation to BF and MT differences. Linear regression analyses of the scatter plots showing the percent inhibition (A), DR, MT and latency shifts (B,C,D) in relation to BF and MT differences. N: number of neurons (see Fig. 6 for legends).

### Data Collection and Analysis

An IC neuron’s response under different stimulation conditions was amplified and band-pass filtered (ISO-DAM, WPI, USA) before being sent to an oscilloscope (TDS210, Tek, USA) and an audio monitor (Grass AM9, USA).The neuron’s response was also sent to a computer (Kaitian 4600, Lenovo, China) for acquisition of peri-stimulus-time (PST) histograms (bin width: 250 µs, sampling period: 150 ms) to 32 sound presentations. The PST histogram showed the neuron’s temporal discharge pattern to sound stimulus. The total number of impulses in each histogram was used to quantify the neuron’s response under each stimulus condition.

**Figure 10:**
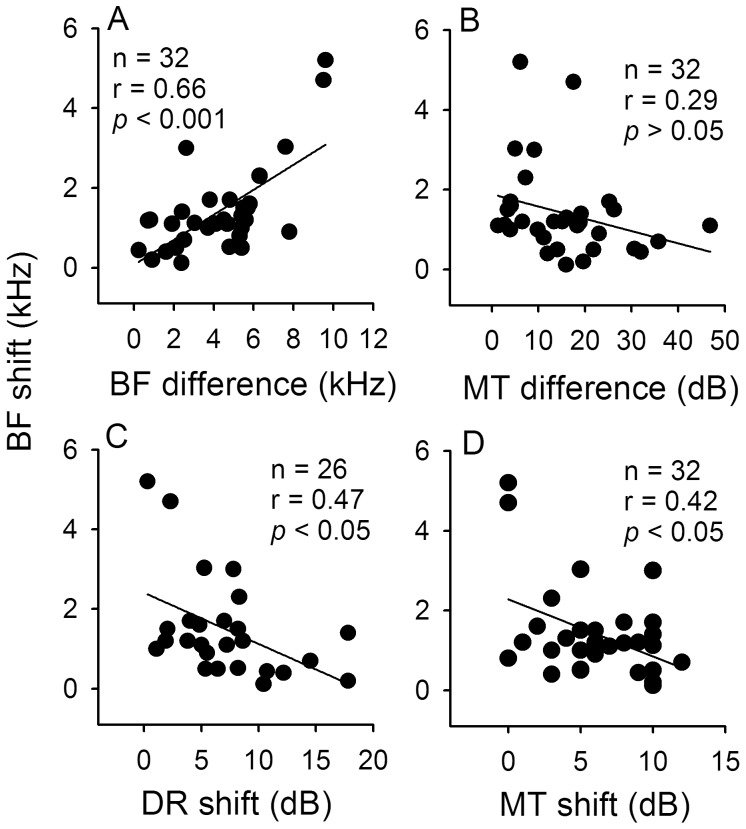
Correlation among different modulated parameters of IC_Mdu_ neurons in relation to BF and MT differences. Linear regression analyses of the scatter plots showing the BF shift in relation to BF and MT differences (A,B) as well as to DR and MT shift (C,D). N: number of neurons (see Fig. 6 for legends).

The modulation of response of each IC_Mdu_ neuron by IC_ES_ focal electrical stimulation was studied by calculating the change in the control number of impulses and dynamic range (DR) of the RLF of the IC_Mdu_ neuron obtained before, during or after IC_ES_ focal electrical stimulation. A DR of a RLF is the range of the stimulus level defined by a neuron’s response magnitude at 10% above the minimum and below the maximum. All the BF and MT differences between IC_ES_ and IC_Mdu_ neuron and the shifts in different parameters of IC_Mdu_ neurons during or after relative to before electrical stimulation are calculated in absolute values. All data obtained under different stimulation conditions were processed and plotted using Sigmaplot 2000. They were then quantitatively examined and statistically compared using SPSS 13.0 (one-way ANOVA at *p*<0.05 and Student’s *t* test at *p*<0.05).

## Results

### Inhibition and Facilitation of Responses of IC_Mdu_ Neurons during IC_ES_ Focal Electrical Stimulation

Focal electrical stimulation in the IC neurons did not appear to affect their acoustically activated responses which recovered to the control level right after the electrical stimulation (Fig. 1Ba vs Ba’; Bb vs Bb’). Among 99 IC_Mdu_ neurons isolated, the responses of 29 neurons were not modulated during IC_ES_ focal electrical stimulation. In the remaining 70 IC_Mdu_ neurons, IC_ES_ focal electrical stimulation produced a decrease in the number of impulses (30–75%, average: 40.1±11%) and an increase in the response latency (0.1–3 ms, average: 1±0.9 ms) of 61 (87%) inhibited IC_Mdu_ neurons (Fig. 1Ca vs Ca’). Conversely, IC_ES_ focal electrical stimulation produced an increase in the number of impulses (34.8–91%, average: 60.2±21.4%) and a decrease in the response latency (0.5–2.5 ms, average: 1.1±0.7 ms) of 9 (13%) facilitated IC_Mdu_ neurons (Fig. 1Cb vs Cb’).

As shown in Table 1, these two groups of IC_Mdu_ neurons did not differ in the recording depth, BF, MT and latency indicating that they are not separately located within the IC. They were tonotopically organized within the IC such that their BF progressively increased with the recording depth (Fig. 2A). However, no correlation was found between the latency and BF or between the BF and MT of these IC_Mdu_ neurons or between the BF and MT differences of IC_ES_ and IC_Mdu_ neurons (Fig. 2B,C,D, *p*>0.05). These findings suggest that IC_Mdu_ neurons in each iso-frequency lamina might have similar BFs but quite different MTs. These findings are in agreement with those reported in previous studies [46]–[49].

Figure 3 shows the distribution histograms of BF and MT differences of these inhibited and facilitated IC_Mdu_ neurons. It is clear that both inhibited and facilitated IC_Mdu_ neurons had higher or lower BF and MT than corresponding IC_ES_ neurons such that their BF and MT differences were bilaterally distributed. Although these bilateral BF and MT differences did not differ significantly (*t* test, *p*>0.05), inhibited IC_Mdu_ neurons had wider distribution of BF and MT differences than facilitated IC_Mdu_ neurons (Fig. 3A-1,B-1 vs A-2,B-2). The BF and MT differences of inhibited IC_Mdu_ neurons were mostly less than 5 kHz (47/61, 77%) and 20 dB (49/61, 80%) while they were all less than 2 kHz and 15 dB for facilitated IC_Mdu_ neurons. As such, the former had larger average BF and MT differences than the latter had (Fig. 3A-3, 2.9±2.5 vs 0.9±0.3 kHz; Fig. 3B-3, 13.5±10.8 vs 6.4±4.1 dB).

The degree of modulation of IC_Mdu_ neurons produced by IC_ES_ focal electrical stimulation varied with the interval between acoustic and electrical stimuli (ISI). As the ISI was systematically varied such that the electrical stimulus first appeared before, simultaneously and then after the acoustic stimulus, the number of impulses of the inhibited IC_Mdu_ neuron decreased from a large number to a minimum at the optimal ISI and increased thereafter with further variation in the ISI (Fig. 4A-1, left ordinate). In contrast, the neuron’s response latency increased from a short latency to the longest one at the optimal ISI before decreasing to a short one again with further variation in the ISI (Fig. 4A-1, right ordinate). The opposite effects were observed for the facilitated IC_Mdu_ neuron. The neuron’s number of impulses increased from a minimum to the maximum at the optimal ISI before decreasing to another minimum with further variation in the ISI (Fig. 4B-1, left ordinate). Conversely, the neuron’s response latency decreased from a long latency to the shortest one at the optimal ISI and it then increased to another long one with further variation in the ISI (Fig. 4B-1, right ordinate). As in the previous study [50], we defined the inhibitory latency that produced the longest response latency at the optimal ISI as the best inhibitory latency (arrow in Fig. 4A-1, abscissa). We also defined the facilitatory latency that produced the shortest response latency at the optimal ISI as the best facilitatory latency (arrow in Fig. 4B-1, abscissa). In this study, the average optimal ISI was 2.1±1.5 ms (range: 0–7 ms) for 61 inhibited IC_Mdu_ neurons and 2.6±2.2 ms (range: 0–8 ms) for 9 facilitated IC_Mdu_ neurons.

Linear regression analyses of the scatters plots of the best inhibitory latency of IC_Mdu_ neurons against BF and MT differences showed a significant correlation of the best inhibitory latency with the BF difference but not with the MT difference (Fig. 4A-2 vs A-3). However, similar analyses were not performed for the 9 facilitated IC_Mdu_ neurons because of the small sample size and narrow range of BF and MT differences (Fig. 4B-2,B-3). The average best inhibitory latency of 61 inhibited IC_Mdu_ neurons was 16.6±5 ms (range: 9–29 ms) which was longer than the average best facilitatory latency of 9 facilitated IC_Mdu_ neurons (13.2±1.9 ms, range: 11.5–17.5 ms) (Fig. 4D).

### The Time Course of Modulation of the RLF of IC_Mdu_ Neurons after 30 Minute IC_ES_ Focal Electrical Stimulation

To determine the time course of modulation of the RLF of IC_Mdu_ neurons, we measured their RLFs at different time frames after 30 minutes of IC_ES_ focal electrical stimulation. As shown in Fig. 5, the representative inhibited IC_Mdu_ neuron had a monotonic RLF in which the neuron’s number of impulses progressively increased with sound level. The neuron’s RLF was decreased to varying degree with sound level resulting in a decreased DR after 30-minute IC_ES_ focal electrical stimulation (Fig. 5A unfilled vs filled circles, DR decreased from 26.5 dB to 17 dB). The decreased RLF and DR slowly returned to the control level (measured before IC_ES_ focal electrical stimulation) over a period of more than 120 minutes. The largest DR shift (decrease) occurred right after the 30 minute IC_ES_ focal electrical stimulation (Fig. 5Ab,C).

Opposite to these observations, the representative facilitated IC_Mdu_ neuron had a non-monotonic RLF in which the neuron’s number of impulses progressively increased with sound level up to a maximum but sharply decreased thereafter at still higher sound level (Fig. 5B). The neuron’s RLF was elevated to varying degree with sound level resulting in an increased DR after 30 minute IC_ES_ focal electrical stimulation (Fig. 5B unfilled vs filled circles, DR increased from 32.7 dB to 41.2 dB). The elevated RLF and increased DR slowly returned to the control level over a period of 90 minutes. The largest DR shift (increase) occurred right after the 30 minute IC_ES_ focal electrical stimulation. Among 31 IC_Mdu_ neurons studied, the recovery time of DR shift produced by 30 minute IC_ES_ focal electrical stimulation was within 30 minutes in 5 neurons, 60 minutes in 9 neurons, 90 minutes in 9 neurons, 120 minutes in 6 neurons and 150 minutes in 2 neurons.

The DR and MT shifts of IC_Mdu_ neurons produced by IC_ES_ focal electrical stimulation did not bear any relationship with the DR and MT of electrically stimulated IC_ES_ neurons. However, linear regression analyses of the scatter plots of the DR and MT shifts and the MT of IC_Mdu_ neurons revealed that the DR and MT shifts as well as the MT shift and MT of IC_Mdu_ neurons were significantly correlated (Fig. 6A,B, *p*<0.05−0.001).

### Level-dependent Modulation of IC_Mdu_ Neurons during IC_ES_ Focal Electrical Stimulation

To study how modulation of amplitude sensitivity of IC_Mdu_ neurons due to IC_ES_ focal electrical stimulation might vary with sound level, we first obtained the RLF of IC_Mdu_ neurons before and during IC_ES_ focal electrical stimulation. We then calculated and compared the percent change in the number of impulses of IC_Mdu_ neurons at each sound level. As shown in Fig. 7, the number of impulses of both inhibited and facilitated IC_Mdu_ neurons increased monotonically with sound level before and during IC_ES_ focal electrical stimulation (Fig. 7Aa,Ba, filled vs unfilled circles). The percent inhibition and facilitation of IC_Mdu_ neurons reduced sharply with increasing sound level (Fig. 7Ab,Bb). On average, the inhibition and facilitation of IC_Mdu_ neurons during IC_ES_ focal electrical stimulation greatly reduced with sound level within 20–30 dB above the MT before reaching a plateau value at still higher sound levels (Fig. 7Ac,Bc).

### Modulation Effect in Relation to BF Difference between IC_ES_ and IC_Mdu_ Neurons

Since collicular neurons are tonotopically organized within the IC (Fig. 2A), we determined if IC_ES_ focal electrical stimulation in one IC produced different degree of modulation of IC_Mdu_ neurons that were located in different frequency laminae of the other IC. In other words, we determined if modulation of IC_Mdu_ neurons by IC_ES_ focal electrical stimulation was related with the BF difference between IC_ES_ and IC_Mdu_ neurons. Representative observations of modulation of six IC_Mdu_ neurons during focal electrical stimulation of two respective IC_ES_ neurons are shown in Fig. 8.

Focal electrical stimulation of one IC_ES_ neuron produced inhibition of two IC_Mdu_ neurons and facilitation of one IC_Mdu_ neuron (Fig. 8A vs B). The BF and BF difference of these three IC_Mdu_ neurons varied systematically with recording depth. Clearly, the percent modulation in the number of impulses was the greatest for the facilitated IC_Mdu_ neuron (85.7%) with a BF difference of 0.8 kHz. The percent inhibition for the two inhibited IC_Mdu_ neurons was larger for the neuron with a smaller BF difference (43.7%, 0.5 kHz) than for the other neuron with a larger BF difference (37.5%, 2.4 kHz). Focal electrical stimulation of another IC_ES_ neuron produced inhibition of all three IC_Mdu_ neurons in which BFs progressively increased with recording depth (Fig. 8C). The percent inhibition was closely correlated with the BF difference (Fig. 8D). The smaller the BF difference was, the greater the percent inhibition became.

To determine if bilateral collicular interaction on amplitude-domain signal processing is correlated with BF and MT differences, we obtained the scatter plots of percent inhibition and DR, MT and latency shifts of inhibited IC_Mdu_ neurons against BF and MT differences (Fig. 9). Linear regression analyses of these plots showed that the percent inhibition, DR and latency shifts is significantly correlated with the BF difference (Fig. 9A-1,B-1,D-1, *p*<0.05−0.001). On the other hand, the DR and MT shift are significantly correlated with the MT difference (Fig. 9B-2,C-2, *p*<0.05−0.001). A similar correlation analysis was not performed for the 9 facilitated IC_Mdu_ neurons due to small sample size.

In this study, IC_ES_ focal stimulation also produced BF shift of IC_Mdu_ neurons toward that of electrically stimulated IC_ES_ neurons when the BF difference was between 2 and 8 kHz (Cheng et al., in preparation). For comparison with a previous study of corticofugal modulation of collicular amplitude-domain processing ([25]; see Discussion), we performed linear regression analyses of the scatter plots of BF shift against BF and MT differences as well as DR and MT shifts (Fig. 10). These analyses revealed that the BF shift significantly increased with BF difference but decreased with DR and MT shifts (Fig. 10A, *p*<0.001; C,D, *p*<0.05). In agreement with the previous study [25], the BF shift is not significantly correlated with the MT difference (Fig. 10B).

## Discussion

### Modulation of IC_Mdu_ Neurons by IC_ES_ Focal Electrical Stimulation

In this study, we used an electrical stimulus of 25 µA to activate IC_ES_ neurons, similar to those used in previous studies [23], [24], [31], [50]. This focal electrical stimulation can effectively activate IC_ES_ neurons without changing their auditory response properties (Fig. 1Ba vs a’; b vs b’). This IC_ES_ focal electrical stimulation respectively weakens and strengthens the effectiveness of a sound stimulus through inhibition and excitation of modulated IC_Mdu_ neurons. As a result, the number of impulses and latency of inhibited IC_Mdu_ and facilitated IC_Mdu_ neurons changed in opposite ways and varied with the ISI (Figs.1C, 4A-1,B-1). The fact that inhibited IC_Mdu_ neurons had larger BF and MT differences than facilitated IC_Mdu_ neurons suggests that bilateral collicular interaction is mediated through wide spread inhibition and focused facilitation (Fig. 3A-3,B-3).

The degree of modulation of IC_Mdu_ neurons produced by IC_ES_ focal electrical stimulation was the greatest at MT level but decreased progressively with sound level (Fig. 7). Conceivably, this observation is due to the fact that bilateral collicular interaction produces a constant amount of inhibitory or facilitatory modulation of IC_Mdu_ neurons at all sound levels and the effectiveness of modulation progressively decreases when the excitation of IC_Mdu_ neurons increases with sound level. This observation is consistent with a previous study that shows that bilateral collicular interaction can mediate both excitatory and inhibitory effects via the commissure of the IC and the greatest modulating effects occurring at near-threshold levels [40]. A similar observation has also been reported in previous studies of corticofugal modulation and forward masking modulation of IC neurons [22], [23], [39], [41], [43], [50].

IC_ES_ focal electrical stimulation compressed the RLF, decreased the DR and increased the MT of inhibited IC_Mdu_ neurons but produced opposite effects on facilitated IC_Mdu_ neurons, the induced shift in MT and DR is significantly correlated (Figs. 5, 6A). Conceivably, the role of bilateral collicular interaction is to sharpen the amplitude sensitivity of inhibited IC_Mdu_ neurons through wide spread inhibition and to enhance responsiveness of facilitated IC_Mdu_ neurons to tuned sound stimulus through focused facilitation. Since 30 minute IC_ES_ focal electrical stimulation also produced a long term shift in DR and MT of IC_Mdu_ neuron, the bilateral collicular interaction may be also involved in acoustic-experience-dependent plasticity in the IC.

We observed that IC_ES_ focal electrical stimulation produced greater MT shifts for IC_Mdu_ neurons with lower than with higher MT (Fig. 6B). This is perhaps due to the fact that IC_Mdu_ neurons with higher MT would require stronger sound for excitation and the modulation effect of IC_ES_ focal electrical stimulation is most effective at low than at high sound level (Fig. 7).

### Modulation of IC_Mdu_ Neurons is BF-difference Dependent

Previous studies indicate that the two ICs have tonotopically appropriate reciprocal connections with each other [12], [14], [17], [51]. This well organized tonotopic organization of both ICs suggests that IC_Mdu_ neurons with small BF differences would receive stronger collicular interaction influences than IC_Mdu_ neurons with large BF differences. In other words, modulation effect produced by IC_ES_ focal electrical stimulation attenuates with distance along the tonotopic axis of the IC. This is supported by our findings that the inhibited IC_Mdu_ neurons with smaller BF differences have shorter best inhibitory latency, larger inhibition and shift in DR and latency than inhibited IC_Mdu_ neurons with larger BF differences had (Figs. 4A-2, 9A-1, B-1,D-1).

We observed that the MT shift produced by IC_ES_ focal electrical stimulation is significantly correlated with both the MT of IC_Mdu_ neurons and the MT difference (Figs. 6B, 9C-2). Also, the BF shift produced by IC_ES_ focal electrical stimulation not only is significantly correlated with the BF difference but also with the DR and MT shift (Fig. 10A,C,D). These observations are quite different from a previous study in bat which shows that BF, MT and DR shift produced by corticofugal modulation is only significantly correlated with BF, MT and DR differences between collicular and cortical neurons, respectively [25], [38]. These differences suggest that corticofugal and bilateral collicular modulation of amplitude signal processing in the IC is complement but not entirely comparable.

### Possible Neural Pathways Underlying the Bilateral Collicular Interaction

What are the possible neural pathways underlying bilateral collicular interaction? As described earlier, each IC receives multiple inputs from many bilateral lower auditory nuclei, the auditory cortex, intrinsic projections within the IC and from the contralateral IC through the commissure of the IC [2]–[17]. Therefore, there are at least three possible pathways that can mediate the bilateral interactions observed in the present study. First, IC_ES_ focal electrical stimulation produces bilateral collicular interaction through the commissure of the IC. Second, IC_ES_ focal electrical stimulation activates the ascending pathways to directly or indirectly excite the ipsilateral auditory cortex which subsequently modulates the response of contralateral IC directly or through the contralateral auditory cortex by way of the corpus callosum. Third, IC_ES_ focal electrical stimulation activates the descending pathways to excite neurons in the lower auditory nuclei which subsequently modulate the response of contralateral IC through multiple ascending neural pathways.

In the present study, we showed that modulation of IC_Mdu_ neurons by IC_ES_ focal electrical stimulation is closely correlated with BF difference (Figs. 8, 9A-1,C-1,D-1, 10A). These findings are nicely corroborated by a recent anatomical study of the topographical organization of the commissural connections between two ICs [51]. This study reveals that commissural neurons in the central nucleus of IC send a divergent projection to the equivalent frequency-band laminae in the corresponding central nucleus of IC and the density of this projection is greatest between corresponding points; consistent with a point-to-point emphasis in the wiring pattern (carton in Fig. 8). Conceivably, this divergent projection from one IC to the frequency-band laminae of the contralateral IC may be the anatomical basis underlying the BF difference-dependent modulation of IC_Mdu_ neurons during IC_ES_ focal electrical stimulation. Because facilitated IC_Mdu_ neurons have smaller BF differences than inhibited IC_Mdu_ neurons have (Fig. 3A-3), the former may be mediated by the more focused point-to-point connections between corresponding frequency laminae in two ICs and the latter may be mediated by the divergent connections between non-corresponding frequency laminae in two ICs. If this is true, the facilitated IC_Mdu_ neurons would have a shorter best affected latency than inhibited IC_Mdu_ neurons had (Fig. 4D).

Previous studies indicate that focal cortical electrical stimulation not only evoke cortical, thalamic and collicular BF shifts but also evoke subcollicular BF shifts [36], [52]. In addition, it has been shown that the collicular BF shift evoked by electrical stimulation of the neighboring collicular neuron is mediated mainly through ipsilateral corticofugal feedback [37]. Therefore, future studies are necessary to determine if bilateral collicular interaction might also be mediated through the corticofugal feedback loop and/or subcollicular pathways. These studies may involve the inactivation of the ipsilateral auditory cortex with Lidocaine and/or by ablation of the commissure of the IC during IC_ES_ focal electrical stimulation.
